# Electromigration-induced directional steps towards the formation of single atomic Ag contacts

**DOI:** 10.3762/bjnano.11.55

**Published:** 2020-04-22

**Authors:** Atasi Chatterjee, Christoph Tegenkamp, Herbert Pfnür

**Affiliations:** 1 Institut für Festkörperphysik, Leibniz Universität Hannover, Appelstraße 2, 30167 Hannover, Germany; 2 Laboratorium für Nano und Quantenengineering (LNQE), Leibniz Universität Hannover, Schneiderberg 39, 30167 Hannover, Germany; 3 Institut für Physik, Technische Universität Chemnitz, Reichenhainer Str. 70, 09126 Chemnitz, Germany

**Keywords:** electromigration, focussed ion beam, nanostructures, silver, Si substrate

## Abstract

Even though there have been many experimental attempts and theoretical approaches to understand the process of electromigration (EM), it has not been quantitatively understood for ultrathin structures and at grain boundaries. Nevertheless, we showed recently that it can be used reliably for the formation of single atomic point contacts after careful pre-structuring of the initial Ag nanostructures. The process of formation of nanocontacts by EM down to a single-atom point contact was investigated for ultrathin (5 nm) Ag structures at 100 K by measuring the conductance as a function of the time during EM. In this paper, we compare the process of thinning by EM of structures with constrictions below the average grain size of Ag layers (15 nm) with that of structures with much larger initial constrictions of around 150 nm having multiple grains at the centre constriction prior to the formation of a point contact. Even though clear morphological differences exist between both types of structures, quantized conductance plateaus showing the formation of single point contacts have been observed for both. Here we put emphasis on the thinning process by EM, just before a point contact is formed. To understand this thinning process, the semi-classical regime before the contact reaches the quantum regime was analyzed in detail. For this purpose, we used experimental conductance histograms in the range between 2*G*_0_ and 15*G*_0_ and their corresponding Fourier transforms (FTs). The FT analysis of the conductance histograms exhibits a clear preference for thinning along the [100] direction. Using well-established models, both atom-by-atom steps and ranges of stability, presumably caused by electronic shell effects, can be discriminated. Although the directional motion of atoms during EM leads to specific properties such as the instabilities mentioned, similarities to mechanically opened contacts with respect to cross-sectional stability were found.

## Introduction

The transition from a three-dimensional (3D) conductor to single atomic chains or atomic point contacts is an intriguing process, which has been addressed many times over the years. Its many aspects ranging from bulk solid-state physics to the stability of various types of clusters, and their attachment to the environment to one-dimensional (1D) properties of atomic chains and contacts have been treated in many different studies [1–4]. However, this topic is not only of pure scientific interest, it is also relevant in the context of the reliable formation of ultrasmall interconnects or contacts of atomic size [5]. The latter topic is particularly challenging, since the exact value of the quantized contact resistance depends explicitly not only on the materials used and their valency [5–6], but also on the shape of the contact [5]. This is the reason why most studies only present histograms of the distribution of measured conductance values, since the exact local geometry at the contacts cannot be controlled.

Properties of metallic contacts of atomic size have been experimentally studied by using techniques such as mechanically controllable break junctions (MCBJ), scanning tunneling microscopy (STM) and electromigration (EM). All these techniques rely on conductance histograms as a statistical tool in order to find the configurations of high stability. Conductance histograms provide information about the most probable conductance values, and their distribution around these values, occuring during the thinning process. Typically, an overall probability distribution of several different measurements is taken that averages out possible instabilities and variations of the individual measurements. Both experiments and theoretical simulations, partly going far beyond the free-electron model, give clear evidence for the existence of quantized conductance in atomic point contacts. The exact conductance values, however, turn out to depend significantly on the local contact configurations so that they may deviate from integer multiples of 2*e*^2^/*h* [5,7].

Furthermore, conductance histograms of alkali metals and the direct comparison of conductance peak values with the magic numbers of cluster size suggest that the preferred electronic quantum modes influence the mechanically stable diameters [8–9]. This electronic shell effect was not only observed for alkali metals, but also in monovalent noble metals such as Ag and Au [10–11]. These experimental findings could be very well correlated with the theoretical simulations of conductance histograms [7,12–13]. The theoretical calculation of conductance histograms is based on the semi-classical interpretation of conductance quantisation proposed by Sharvin [14], where conductance is essentially proportional to the contact area [5,15].

In mechanical stretching experiments, real-time HRTEM investigations [16–17] showed the thinning of preferred crystallographic orientations towards the formation of point contacts. But such studies are difficult in case of EM experiments. There were attempts to record real-time SEM and TEM of EM junctions [18–20], but these imaging studies did not observe the conductance states at the semi-classical range just prior to point contact formation. In our study, the thinning mechanism of EM at grain boundaries correlated with electrical conductance histograms adds additional information to the existing studies just mentioned.

The understanding of the origin of conductance histogram peaks can be deepened by searching for correlations between conductance values in the histograms. This information is obtained from Fourier transforms (FTs) of the conductance histograms. It also contains information about the structural thinning process, as demonstrated previously for several metallic systems [21–24], since, depending on the metal (fcc or bcc structure), the calculated ratios of frequencies in the FTs were compatible with a preferential growth in certain crystallographic high-symmetry directions. Our study also uses these tools for data analysis.

We think that our study presented below sheds light on several aspects of EM not considered in detail previously. Contrary to most EM experiments with thin metallic films on insulating substrates, the Ag/Si(100) system is unique in the sense that the first Ag layer wets the hydrogen-terminated Si(100) surface [25]. This improves the thermal contact so that thermally assisted processes during EM can be suppressed to a large extent, in agreement with our own simulations [26]. This situation differs from most previous EM experiments, in which it was difficult to separate EM from thermal effects. For our experiments we use ultrathin Ag films (thickness 5 nm), which exhibit Stranski–Krastanov growth behavior so that they are nanocrystalline with an average grain size between 30 and 50 nm.

These grain boundaries turned out to be the main source of lateral resistance [27]. Therefore, the EM-induced material transport is mainly expected to take place at these boundaries. EM at grain boundaries, studied here in detail, is less well defined than in single-crystalline materials. Therefore, the question arises whether a well-defined growth direction can be identified at all. Our answer is positive.

Furthermore, we were recently able to demonstrate very different morphologies upon EM of such films depending on the size of the smallest constrictions. For bow-tie structures with a smallest constriction of typically 150 nm, generated by standard e-beam lithography, we observed the EM-induced formation of filamentous structures at a surface temperature of 100 K. A single electrically conducting path could not be identified visually nor be reproducibly generated. This contrasts with experiments where the smallest constriction was reduced by one order of magnitude down to about 15 nm using a focused ion beam (FIB), i.e., far below the average grain size in the Ag film, in which complex morphological changes were absent [28].

Thus, we have a well-defined reference system generated by EM. Therefore, it seems meaningful to obtain more details about the thinning process induced by EM in this system from the information contained within the experimental conduction histograms and their FTs. Furthermore, since the morphological appearance of the EM-induced structuring process for the large structures appear to be fundamentally different, such a study could also clarify whether these differences also appear in the conduction histograms and their FTs.

## Results and Discussion

**Figure 1:**
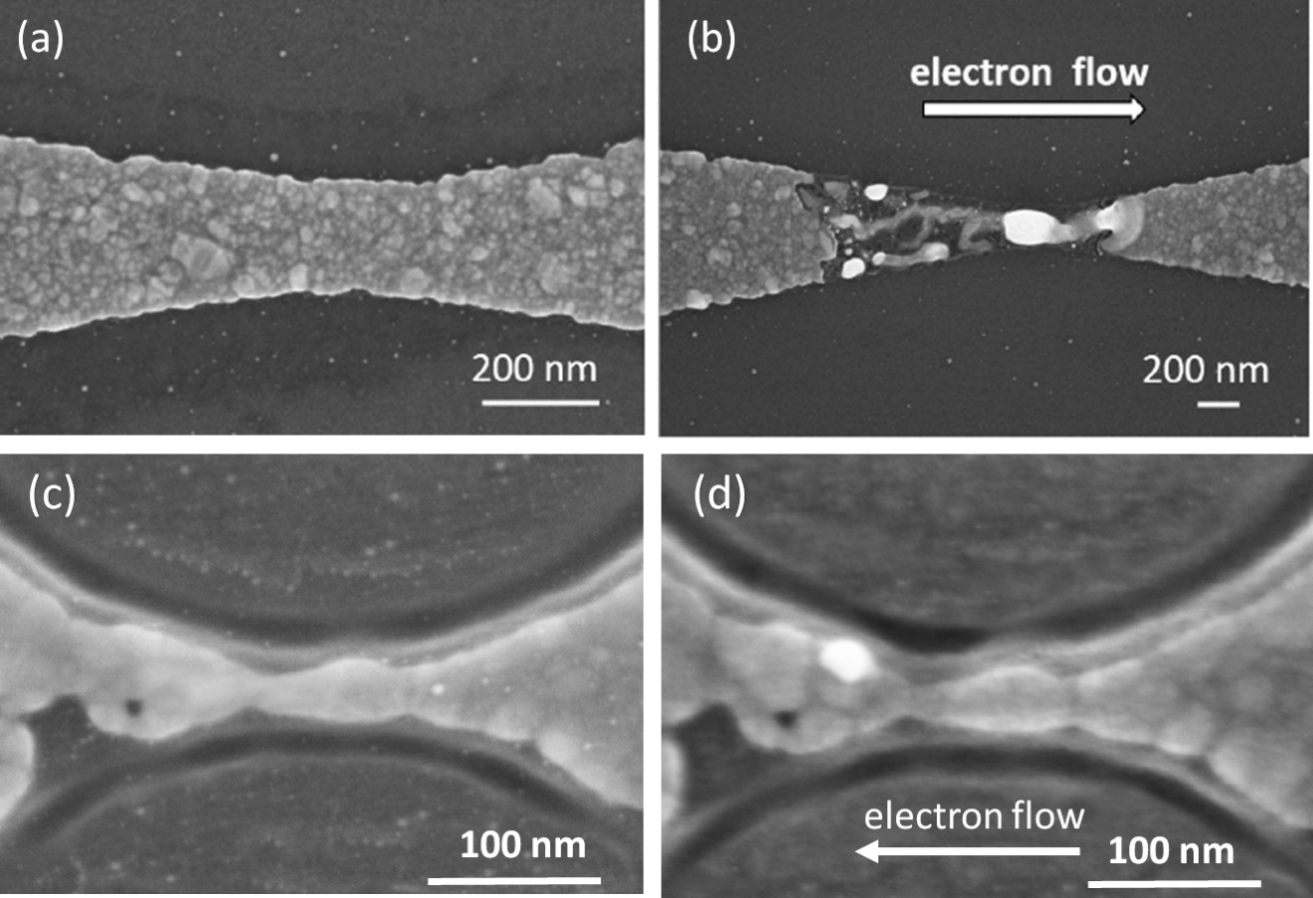
(a) Typical SEM image of a 5 nm thick, nanocrystalline Ag bow-tie structure before EM and (b) after EM yielding a conductance value of around 1*G*_0_. (c) SEM of a further FIB-patterned bow-tie structure before EM with elliptical grooves reducing the centre constriction to 17 nm before EM and (d) after EM yielding a conductance value of 1.3*G*_0_. Please note the different scale bars. Panels (c) and (d) reproduced permission from [28], copyright 2018 AIP Publishing.

In order to illustrate the importance of ultra-narrow structuring for obtaining reliable results, we present SEM images of Ag nanostructures before and after EM for bow-tie structures with a centre width between 100 and 200 nm in comparison with FIB-patterned bow-tie structures with a centre width below 20 nm (see Figure 1). EM in the wide Ag contact results in clear unidirectional material transport, as seen by the large clusters preferentially formed on the right-hand side of Figure 1b, appearing as white spots. However, a filamentous structure is always formed on the left-hand side, which neither allows one to identify the exact location of the point contact nor any reproducible production of point contacts. Nevertheless, quantized conductance plateaus as a function of time were still observed for these bow-tie structures during EM.

It turned out that the existence of several grains in the cross section of these Ag structures is the reason for this morphological behaviour. Since EM mainly occurs at the grain boundaries, the contact resistance between various grains has a comparable value due to similar sizes of grains and contact areas. Thus, a complicated parallel EM process involving many grains sets in. Material exchange between many of them leads to the formation of filament-like structures with, as far as we can judge, larger grains than before EM. However, since EM is a process with partial positive feedback, also thinning takes place, but the location cannot be well controlled. Nevertheless, after a competition of several grains in the narrowest constriction, a point contact is formed in one of these filaments, which is hard to locate structurally. Electrically these structures exhibit well-defined conductance quantization.

For a much better control of the EM process it turned out that it is sufficient to reduce the number of grains at the centre to one [28]. In this case, the current density is clearly highest at only one grain boundary so that the thinning process happens mainly there, as demonstrated by a comparison between Figure 1c and 1d. For these very narrow structures we obtained highly reproducible values of final conductance in about 95% of the structures investigated.

We now want to address the question how the thinning process in these morphologically quite different structures proceeds under conditions of EM and at temperatures at which thermal diffusion is largely suppressed [26]. Due to the high probability of electron scattering at grain boundaries, material transport mainly happens at and across grain boundaries, but not within the homogeneous crystalline material that is typically assumed in most models. Therefore, deviations from these models must be expected. Focussing for the moment on a single grain boundary, the directed material transport in EM will cause thinning of one grain while the other grain has to take up the material. Thus a strong asymmetry is introduced that is absent in the case of MCBJ experiments, so that these two types of experiments may yield different results. Furthermore, we will show that the chosen starting conditions (bow-tie and FIB-patterned bow-tie structures), which result in the formation of significantly different structures during the EM process, finally undergo similar steps of thinning when getting close to the quantum regime. In order to avoid the pure quantum regime and to understand the mechanism during thinning, we concentrate only on the semi-classical region. Therefore all the conductance histograms discussed here start at 2*G*_0_.

**Figure 2:**
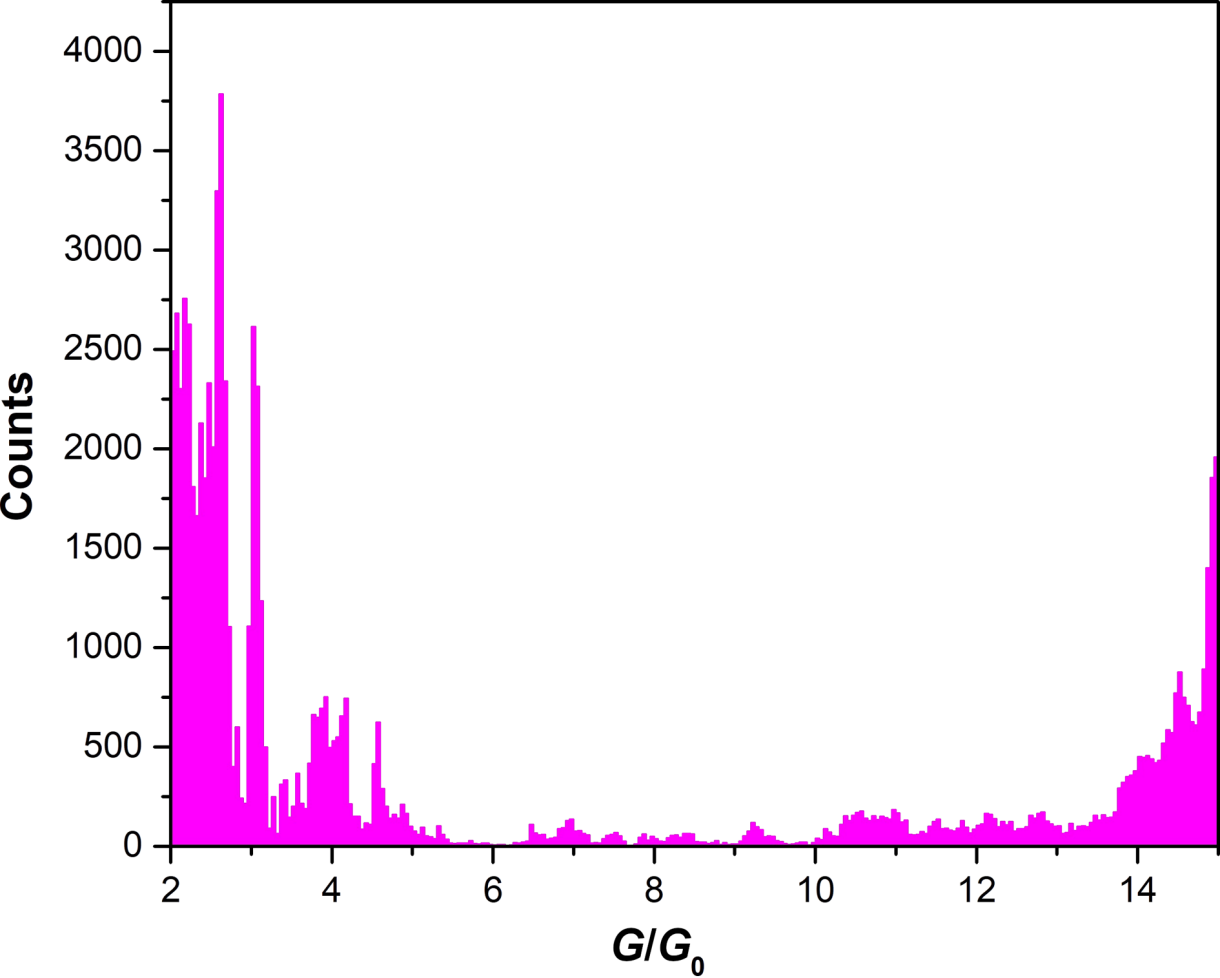
Conductance histogram with values between 2*G*_0_ and 15*G*_0_ of conductance traces obtained during EM-induced thinning from 20 different samples of 150 nm wide structures of bow-tie shape. Bin size: 0.05*G*_0_.

A conductance histogram obtained from the conductance traces during EM of bow-tie structures between 2*G*_0_ and 15*G*_0_ is depicted in Figure 2. Peaks at 2.1*G*_0_, 2.6*G*_0_, 3.0*G*_0_, 3.8*G*_0_, 4.2*G*_0_, 4.6*G*_0_, 14.5*G*_0_ and 15*G*_0_ are observed. At this point it is not clear whether these values are the result of several contacts in parallel or stem from a single contact, since non-integer values of conductance are commonly observed also for single contacts [24,29–30], mainly due to asymmetric and slightly irregular shapes of the contact, in agreement with theoretical simulations [7].

It is remarkable that, between 4.5*G*_0_ and 14*G*_0_, there is a large range of instability, i.e., once the critical conductance falls below 14*G*_0_, further EM barely yields stable configurations until values below 5*G*_0_ are reached. This large range of instability again indicates either a break-up of several contacts (*G >* 15*G*_0_) into a single contact or an instability of a single contact. The first scenario is not very probable. Since about 20 separately generated structures were used and averaged, which have various starting geometries and a different number of conducting channels at large *G*, it is not plausible to expect an instability at the same overall *G* value for all of them. Therefore, we conclude that already at values around 15*G*_0_ it is essentially only one junction that is conducting. Such instabilities seem to be characteristic to the EM process, since they are commonly not observed in MCBJ experiments but have also been found in recent EM experiments with Cu nanocontacts [24]. Since a distribution of contacts of various sizes exist, there is still a small probability for conductance through more than one channel that is reflected by the small number of counts in the range between 14*G*_0_ and 5*G*_0_.

**Figure 3:**
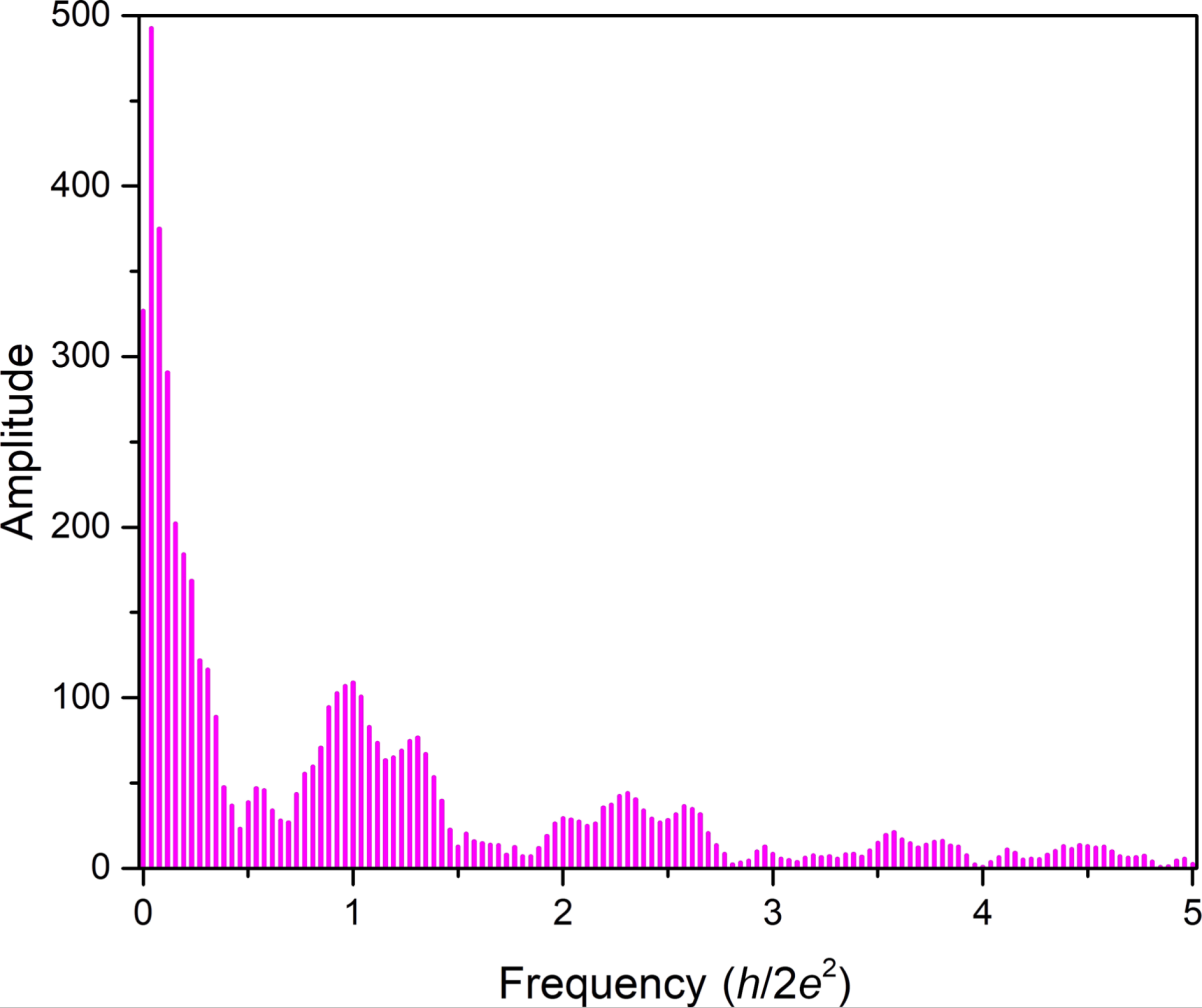
Fourier transform of the conductance histogram of Figure 2 of bow-tie electromigrated structures. The *x*-axis represents the Fourier frequencies while the amplitudes of the frequencies are plotted on the *y*-axis.

After performing a Fourier transform (FT) of this conductance histogram (see Figure 3), a distinct peak structure is observed, which corresponds to characteristic decrements of conductance. It can be interpreted by the semi-classical Sharvin formula. This formula is an approximation for contacts approaching the ballistic regime. Within this model, the nanowire conductance for a circular cross-sectional area, *A*, is given by [21]:

[1] $$g=\frac{G}{G_{0}}=\pi A-{\left(\pi A\right)}^{1/2}+1/6,$$

[2] $$G=gG_{0}=G_{0}\left[{\left(\frac{k_{\text{F}}R}{2}\right)}^{2}-\frac{k_{\text{F}}R}{2}+1/6\right],$$

with the Fermi wavelength λ_F_ = 2π/*k*_F_. In Equation 2 the cross-sectional area *A* is expressed in units of λ_F_^2^. Taking into account the spill-out of electron density beyond the rectangular potential assumed in Sharvin’s model, the two last terms in Equation 1 and Equation 2 nearly cancel out [21]. This leads to a linear relationship between *A* and *g* (Δ*g* = πΔ*A*).

If we ignore for the moment the different orientation of grains – for a justification, see below – and assume that only a single contact is thinned at a time, we can use a previously developed argumentation [21,31] about the preferential direction of thinning. Taking into account the known fcc stacking of Ag in the direction perpendicular to the three principal directions [111], [100], and [110], 2-dimensional (2D) contact areas and their conductance can be identified. The area of the 2D (111), (100), and (110) unit cells is 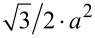, *a*^2^ and 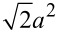 respectively. Here *a* is the lattice constant. If a one-by-one atom decrement of the contact areas of a crystalline grain is considered, the conductance steps have different sizes that scale with Δ*g*_111_:Δ*g*_100_:Δ*g*_110_ = 0.87:1:1.41 for thinning along these directions. Using *k*_F_ of bulk Ag, the calculated periods in the three principal directions correspond (in units of *G*_0_) to Δ*g*_100_ = 0.96, Δ*g*_111_ = 0.83, Δ*g*_110_ = 1.36. The inverse conductance values should appear in the FT of a conductance histogram as (Δ*k*_F_*R*)^−1^, where the spacing between *G* values corresponds to a specific direction. The frequencies obtained from Equation 1 and Equation 2 for an fcc crystal structure are 0.8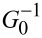, 1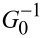 and 1.3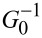 for the three principal crystallographic directions [110], [100] and [111], respectively [21].

In order to apply this theory to the thinning at grain boundaries, we have to recall two facts. Firstly, in nanocrystalline elemental materials like Ag, grain boundaries occur mostly because of the different orientation of nanocrystals. Since the elastic strain energy strongly increases with angular misfit, small-angle grain boundaries are the most likely ones. Thus, most contact areas are not far from (stepped) high-symmetry crystal planes. Secondly, due to its high directionality, EM thins one grain while depositing the material on an adjacent grain. Therefore, the local electrical resistance is determined by the contact area between the grain that is thinned and the adjacent grain that is taking up the material. Only this cross section and its variation by EM is considered. Thus, deviations due to unknown step densities and local strain are ignored when considering only high-symmetry directions of the interface, as we do in the following.

Figure 3 represents the FT of the conductance histogram in Figure 2 of bow-tie structures between 2*G*_0_ and 15*G*_0_. The most dominant frequencies are 1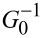 and 1.3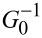. Other peak frequencies in Figure 3 are at 0.6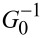, 2.1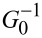, 2.3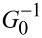 and 2.6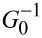. The large peaks below 0.2*h*/2*e*^2^ are characteristic of large jumps in the conductance histograms, as already pointed out in Figure 2. They again denote in part the instability of intermediate conductance values between 14*G*_0_ and 5*G*_0_.

The dominant frequencies at 1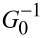 and 1.3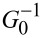 in Figure 3 agree within error bars quantitatively with those derived above for atom-by-atom thinning [21] in the [100] and the [111] direction during EM. Within this argumentation, it is also interesting to see that the contribution from 0.8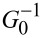, i.e., thinning in the [110] direction, is absent in these structures. This result contrasts with a MCBJ experiment using Au nanowires [21], in which all three frequencies were obtained. It matches, however, with the findings of mechanical stretching experiments of Ag nanowires, observed with HRTEM [32], where it was reported that Ag mostly forms rod-like structures along the [110] direction, which are unable to form wires. Atomic chains turned out to form only when at least one grain was oriented in the [100] direction. The dominant peak at 1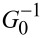 in Figure 3 indeed indicates thinning in this particular direction. From these dominant peaks in the FT and the HRTEM results [32], we conclude that the relevant structures in the conductance window considered here consist preferentially of single junctions that make contact either in the [100] or the [111] direction.

The frequency at 0.6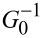 has also been observed before by Mares et al. [10]. It was attributed to relatively stable cross sections due to the formation of diametric orbits. This frequency was found to be very prominent for Ag, less prominent in Cu and absent in Au as observed by the authors of [10]. Correspondingly, the very interesting feature of the 1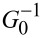 peak is the superposition of square and triangular orbits [5,10].

The frequencies between 2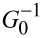 and 3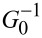 contain clearly the overtones of those frequencies discussed above with prominent peaks at 2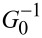 and 2.6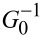, but also with a small peak at 2.3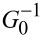, which does not fit into the simple picture. These are contributions from the spacings of metastable configurations with changes of conductance at the sub-*G*_0_ level due to local changes in the close environment of the actual contact. Such sub-*G*_0_ spacings between conductance values can be clearly spotted in the conductance histogram in Figure 2 and have also been observed in simulations of Ag nanocontacts [7].

**Figure 4:**
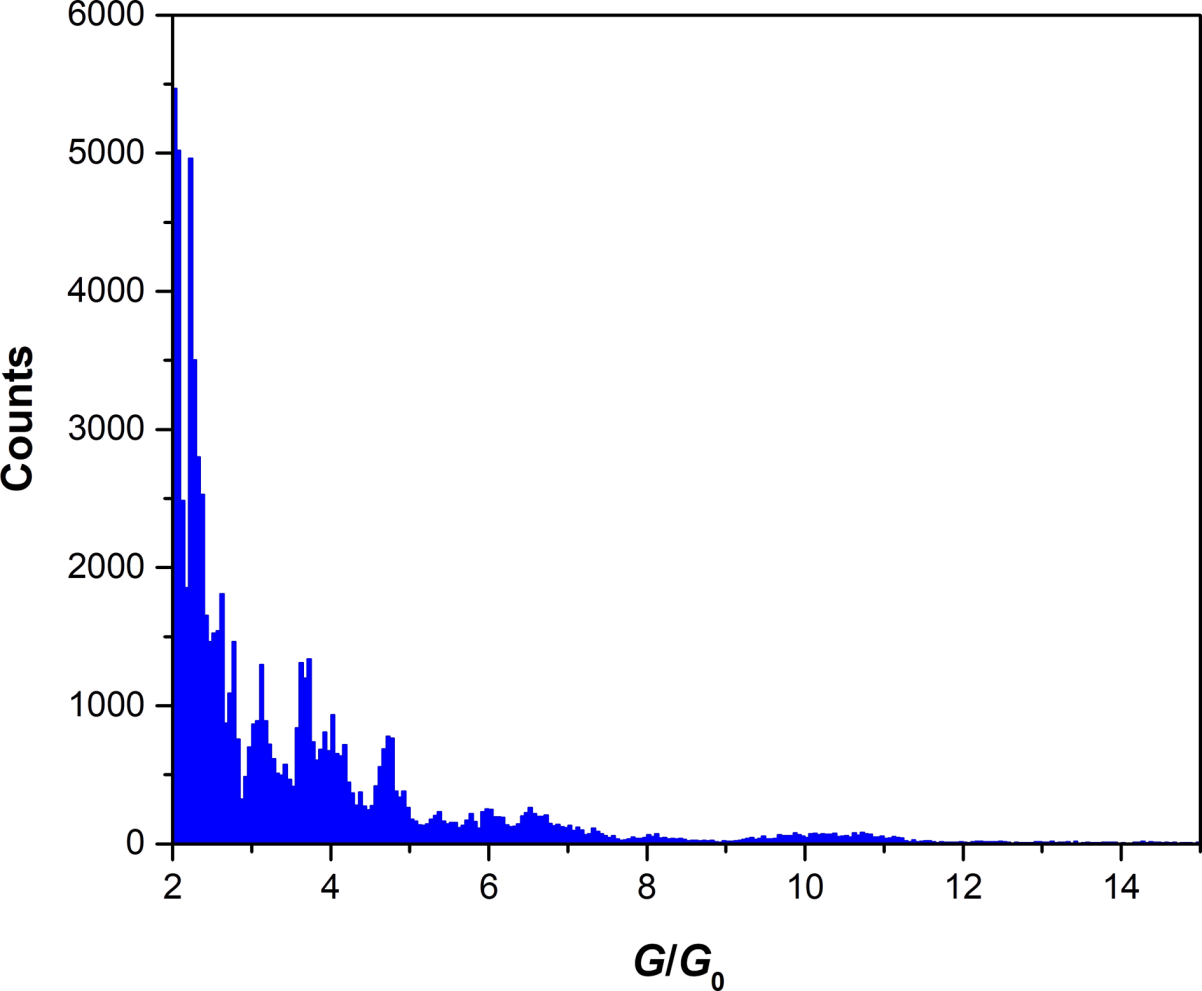
Conductance histogram of 15 conductance traces obtained during EM of FIB-patterned bow-tie structures. Bin size: 0.05*G*_0_.

The results of the FIB-patterned bow-tie structures corroborate the assumptions made above that essentially a single contact was measured already starting with mesoscopic bow-tie structures. The conductance histogram for the FIB-patterned bow-tie structures for the same range of *G* as in Figure 2, is shown in Figure 4 using the average of 15 conductance traces.

A quite similar peak structure as in Figure 2 is observed here between 2*G*_0_ and 5*G*_0_. There are strong peaks at 2.1*G*_0_ and 2.3*G*_0_ but less intense peaks at 2.6*G*_0_ as compared to Figure 2. Moreover, in Figure 4 fine-structure peaks at 2.7*G*_0_, 2.8*G*_0_, 3.2*G*_0_, 3.7*G*_0_, 4*G*_0_ and 4.7*G*_0_ are observed. However, the peaks around 14.5*G*_0_ and 15*G*_0_ are absent in Figure 4, i.e., the range of unstable cross sections is even more extended in this case. This difference may be due to the size distribution of grains, which smears out the range of instability, whereas the results summarized in Figure 4 were obtained from single grains in the starting configuration. In this situation, there is less possibility for a particle exchange between different grains that may reduce the range of visible instabilities.

**Figure 5:**
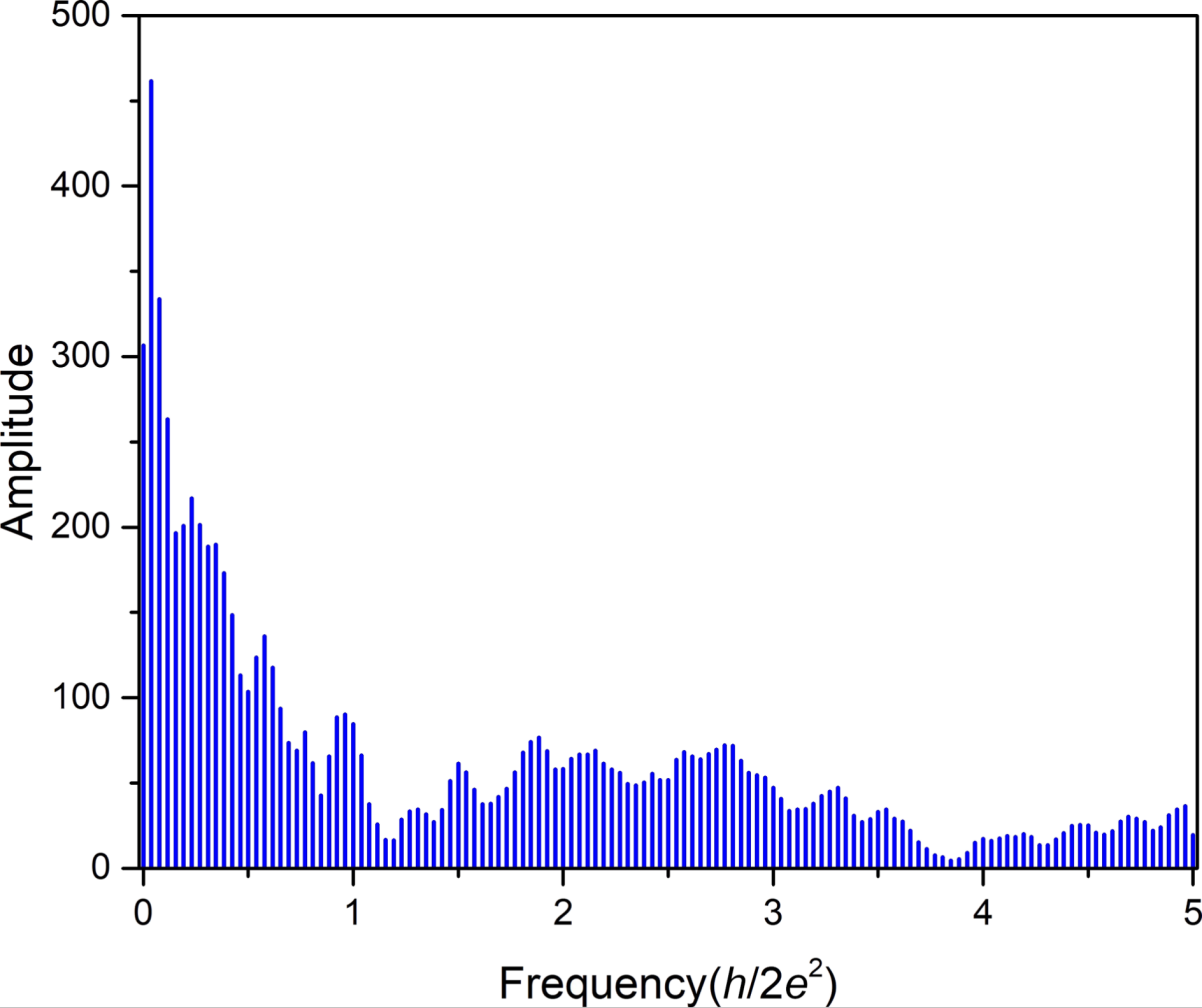
FT of conductance histogram of FIB-patterned structures shown in Figure 4. The *x*-axis represents the Fourier frequencies whereas the amplitudes of the frequencies is plotted in the *y*-axis. The prominent peak at 1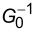 suggests thinning in [100] direction.

The FT of Figure 4, which is shown in Figure 5, qualitatively resembles the FT of Figure 2 shown in Figure 3. This supports our hypothesis that also in the large bow-tie structures we observe only thinning of a single grain once conductance has been reduced to values below 15*G*_0_. Nevertheless, Figures 3 and 5 are not identical. Instead, they show several quantitative similarities and differences.

The first similarity is the peak position at 1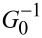, showing that in both cases preferential thinning at [100]-oriented grain boundaries occurs. This dominance of the peak at 1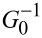 in both types of structures not only gives evidence that the atomic point contact thinning occurs at the [100]-oriented interface, but also demonstrates the prominence of electronic shell effects [10] in these ultra thin Ag films at 100 K. A further similarity with Figure 3 is the presence of the peak at 0.6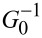. Strong peaks below 0.5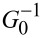 again correspond to instabilities between other metastable configurations.

On the other hand, a noticeable difference between Figure 3 and Figure 5 is that the contribution from the [111] orientation is absent in Figure 5. There is no peak above noise level at 1.3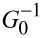. Since FIB structuring is not expected to be selective with respect to the grain orientation, this finding proves that material exchange occurs only along the [100] direction once only a single grain is the starting point of EM. Assuming that the [100] thinning direction is the energetically most favorable one, the [111] direction is only observed when the reduction of conductance from multiple grains to a single grain involves directional changes of the current so that the less likely [111] direction still appears. This is exactly what is seen in Figure 3, whereas the [110] direction was never observed.

Interestingly, another difference in the peak structure between 1.5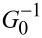 and 3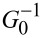 in Figure 5 is that the higher frequencies are somewhat more extended and more pronounced than in Figure 3. While the reasons for its occurrence are similar to those mentioned in context with the latter figure, the histogram of Figure 4 exhibits finer peak spacings in comparison to Figure 2, which gives a different weight to the overtones between 1.5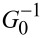 and 3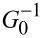 in Figure 5.

## Conclusion

The EM process in ultrathin nanocrystalline Ag structures on Si(100) was investigated for structures that had a narrowest constriction of 100 to 150 nm. These were compared with those further structured down using FIB to 15 nm, i.e., below the size of a single grain. Although the mesoscopic evolution of structures with filament formation for the large structures was very different from that of the structures that were initially only 15 nm wide, the similarity of conductance histograms below 15*G*_0_ lead us to the conclusion that only a single contact existed in most cases. A large range of unstable configurations between 14*G*_0_ and 5*G*_0_ may be characteristic for the EM process at a temperature where only limited thermal diffusion is possible, since such a range of instability was not found in experiments with other techniques. At this point, due to the limited available data set involving only Ag contacts, it remains unclear how general this phenomenon is. It may, however, be related to the observed instability of other thinning directions for Ag.

Although the thinning mechanism of EM seems to be quite different from that of mechanical stretching, we conclude from our FT analysis that the underlying atomistic processes seem to be quite comparable. Similar conclusions are drawn in [33]. This similarity can be rationalized from the fact that, although EM is directional, and, therefore, generates asymmetric contacts, only the narrowest constriction plays a crucial role, so that the exact shape of the contact is comparatively unimportant. A detailed investigation, considering the FTs of conduction histograms, revealed a preference for atom-by-atom thinning along the [100] direction and a combination of geometric and electronic shell effects [21].

This study thus complements existing data from MCBJ measurements of Ag and HRTEM investigations on Ag point contacts and provides a concrete information on the mechanism of thinning in ultrathin Ag films.

## Experimental

Low-doped Si(100) substrates (1000 Ω·cm at 300 K), which are good insulators at temperatures around 100 K, were used. Structuring was carried out by a three-step process. As a first step, we patterned the contact pads by photolithography. Secondly, electron beam lithography was employed in order to obtain nanostructures of bow-tie shape that were 100 to 200 nm wide at the smallest constriction. After HF dip, in order to get a hydrogen-terminated surface, 1 nm of Ti served as wetting layer before we evaporated 5 nm of Ag onto the substrate at room temperature. Thirdly, these bow-tie structures were further patterned by using a FIB in order to reduce the centre width below the size of a single grain. By writing elliptical structures into the Ag nanostructures, we were able to reduce the centre width of the nanostructures to below 20 nm. The detailed steps of the sample fabrication were reported in a previous publication [26,28].

All measurements were performed within a four-tip SEM/STM UHV chamber (base pressure 2 × 10^−10^ mbar). This facilitated cooling of the structures down to 100 K without any spurious condensation. Furthermore, the UHV environment was important for the Ag structures as they are quite susceptible to sulfur contamination under ambient conditions. UHV also provided an ultra-clean environment for point contact measurements. Two out of the four available tips were used for the EM measurements. The tips were pre-cooled by making electrical (and mechanical) contact with the contact pads produced by photolithography.

To perform EM measurements, an in-house LabVIEW program was developed (following Motto et al. [34]), which allowed for a precise control of the conductance in order to obtain atomic point contacts. Suitable feedback parameters and ramp speeds for the applied bias voltage were selected in the program which consisted of two feedback loops. The starting resistance of the structures was typically between 50 and 100 Ω. When the resistance change between two consecutive measurements was less than the preset value, the ramp voltage was increased. In the other case, the control went to the second loop, where momentary resistance changes (due to structural changes) were compared with preset feedback parameters with a response time of 10 ms. Abrupt changes in resistance took place at current densities of (5 ± 2) × 10^13^ A/m^2^ and at voltages between 0.8 and 1.5 V, depending on the actual structure.

Conductance traces were obtained during EM thinning, which demonstrated step-like conductance plateaus. Details of point contact characterisation can also be found in our earlier publication [28]. Conductance histograms constructed using these plateaus revealed the most probable (and temporally stable) conductance values as peaks. Finally a FT analysis of these experimental conductance histograms was performed to identify the crystallographic contributions of the metallic structure.
